# Management of delayed-onset skin flap complications after pediatric cochlear implantation

**DOI:** 10.1007/s00405-020-06348-2

**Published:** 2020-09-11

**Authors:** Qingling Bi, Zhongyan Chen, Yong Lv, Jie Luo, Naya Wang, Yuan Li

**Affiliations:** 1 Department of Otolaryngology, China-Japan Friendship Hospital, Yinghuadong Street, Chaoyang District, Beijing, 100029 China; 2 Department of Pathology, China-Japan Friendship Hospital, Beijing, 100029 China

**Keywords:** Cochlear implant, Flap complication, Revision surgery, Temporalis myofascial flap

## Abstract

**Purpose:**

To review delayed-onset skin flap complications associated with pediatric cochlear implantation (CI) in our institute, analyze the etiology, and explore effective treatment strategies.

**Methods:**

Retrospective analyses of 811 children who had undergone cochlear implantation between January 2003 and March 2019 were performed. Twelve (1.48%) patients developed skin flap complications after CI. We present a classification of flap issues and wound histopathology following cochlear implantation. The interventions for flap problems included drug treatment, aspiration, local wound care, revision surgery, and explantation depending on the clinical situation. The temporalis myofascial reconstructive option is discussed.

**Results:**

Seven subjects were cured with conservative treatment. Five cases with flap infection or necrosis underwent revision surgery, with wound closure in three cases (60%) and revision surgery with explantation in the remaining two cases (40%). Explantation ultimately led to wound healing in all cases. They all achieved excellent performance through re-implantation.

**Conclusion:**

Flap complications after CI are rare but treatable. Comprehensive treatments should be developed to achieve a stable and healed wound for CI.

## Introduction

Cochlear implantation (CI) is the most effective technique for treatment of bilateral severe to profound sensorineural hearing loss. Since the 1960 s, almost 1 million patients have benefited from CI, and it is generally a safe procedure in experienced hands. However, as with all surgical procedures, there are complications associated with CI. The complications depend on patient condition, the type of implant device, and the surgical technique. Skin flap issues are the most common complications associated with CI. The frequency of skin flap complications reported in the literature varies widely from 1.08 to 8.2%. [1–3] However, the definition of flap issues after CI varies between different centers. Wound infection, flap swelling, delayed-onset seroma or hematoma, skin infection, and flap necrosis have been discussed in the literature, but no systematic classification is available. [3] Loundon et al. [4] suggested that scalp lesions are theoretically more likely to occur in children, with increased risk at a younger age. The thinness of the skin, high probability of trauma, and upper respiratory infection can lead to injury of the scalp. Most perioperative flap complications are hematomas and wound infections [1, 3, 5] that are usually simple to detect and resolve. Terry et al. systematically reviewed 88 articles including a total of 22,842 patients; delayed complications after CI were evaluated. The incidence rate of skin infection was 1.3% (238/17,878), compared to 0.9% (48/5486) for hematoma or seroma and 1.0% for device rejection 1.0% (16/1664). [3] Although delayed flap issues are rare, when they develop, the timing and manner of treatment are critical in terms of implant retention. Here, we present retrospective analyses of patients with delayed-onset flap complications following CI and present our experience in the management of different flap problems.

## Materials and methods

### Patients, surgical technique, and follow-up

In total, this retrospective study included 811 children who underwent CI between January 2003 and March 2019. All the operations were performed by the same senior surgeon using a standardized surgical technique. A C-shaped minimal access incision and flap were used in 776 cases. During initial surgery, based on the receiver dimensions of the Med El Combi 40^+^ cochlear implant, extended post-auricular S-shaped incisions were created for 45 subjects in whom the Combi 40^+^ was used (Table 1). A bone well was created to accommodate the receiver-stimulator of the device, which was appropriately fixed. The wound was closed in three layers, i.e., the fascia periosteum, the subcutaneous layer, and the intradermal layer, with 3–0 silk continuous sutures. An elastic head bandage was placed on the head and maintained for 1 week following the operation. A broad-spectrum antibiotic that can cross the blood–brain barrier was provided intravenously during the operative period and hospital stay.

**Table 1:** Implant devices placed in children who had undergone cochlear implantation (*N* = 811)

Device type				Delayed-onset flap complications		
Manufacturer^a^	Total	Model	Cases	No	Yes	
					Cases	Occurrence rate (%)
Cochlear Nucleus	453	CI24RE (ST)	26	449	4	0.88
		CI24RE (CA)	233			
		CI422	6			
		CI512	177			
		CI522	11			
Advanced Bionics	147	HiRes 90 K 1 J	121	142	5	3.4
		1 J Advantage	23			
		HiFocus Midscala	3			
Med El	174	Pulsar	14	171	3	1.72
		Sonata	103			
		Concerto	12			
		Combi 40	45			
Nurotron	37	CS-10A	37	37	0	0
811				799	12	1.48

^a^Advanced Bionics (Advanced Bionics Corporation, Valencia, CA, USA); Cochlear Nucleus (Cochlear Ltd., Sydney, Australia); Med El (Med El Corporation, Innsbruck, Austria); CS-10A (Nurotron Ltd., Zhejiang, China)

All patients underwent long-term follow-up (minimum of 8 months); the longest follow-up was 17 years. Patients who experienced flap complications were examined with regard to age, sex, age at operation, possible causes, onset time, type of implant device, management strategy, and outcomes. A detailed medical history should be taken when assessing the severity of flap-related complications. Flap-related issues that occurred in the perioperative period were excluded from the analyses.

Approval from the institutions’ ethics committees was obtained for this study.

### Classification of delayed-onset flap complications

**Fig. 1:**
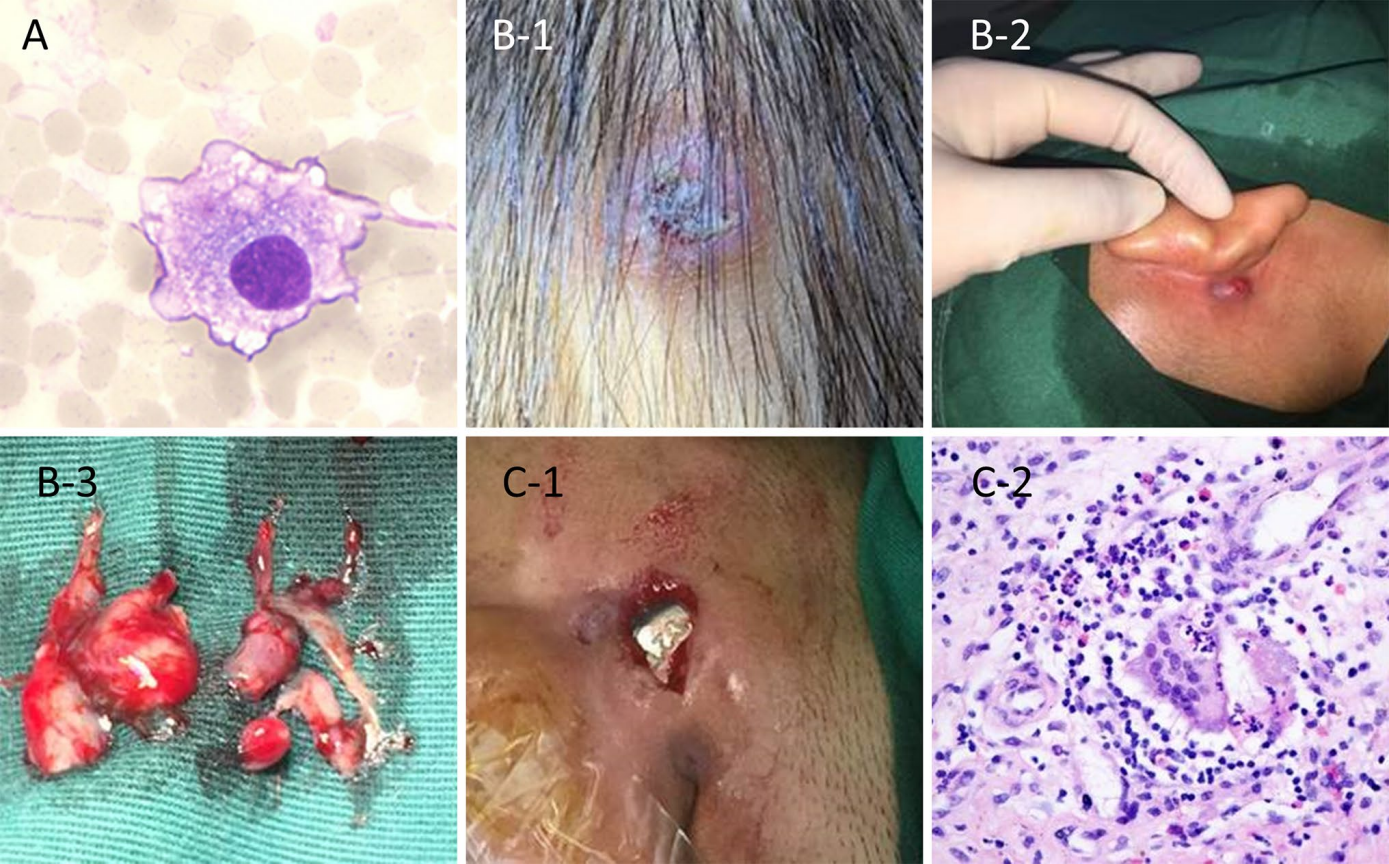
**a** The smear microscopy test showed full-field erythrocytes and a small number of macrophagocytes (HE × 400). **b**-1 A type B patient (#10) who developed swelling of the scalp and an ulcer after exposure to a strong magnetic force. **b**-2 and 3 were type-B cases, with a skin flap infection or necrosis associated with granulation tissue over the wound. **c**-1 Severe skin flap necrosis with implant exposure. **c**-2 (HE × 400) Histopathological findings confirmed infected tissue with infiltration by numerous neutrophils, multinucleated giant cells and a few eosinophil granulocytes

Flap complications that occurred after CI were classified into three types according to severity: (A) flap seroma or hematoma around the receiver-stimulator, (B) skin flap infection or necrosis with development of granulation tissue over the wound, (C) skin flap rupture with implant exposure and extrusion (Fig. 1).

### Therapeutic options for delayed-onset flap complications

**Fig. 2:**
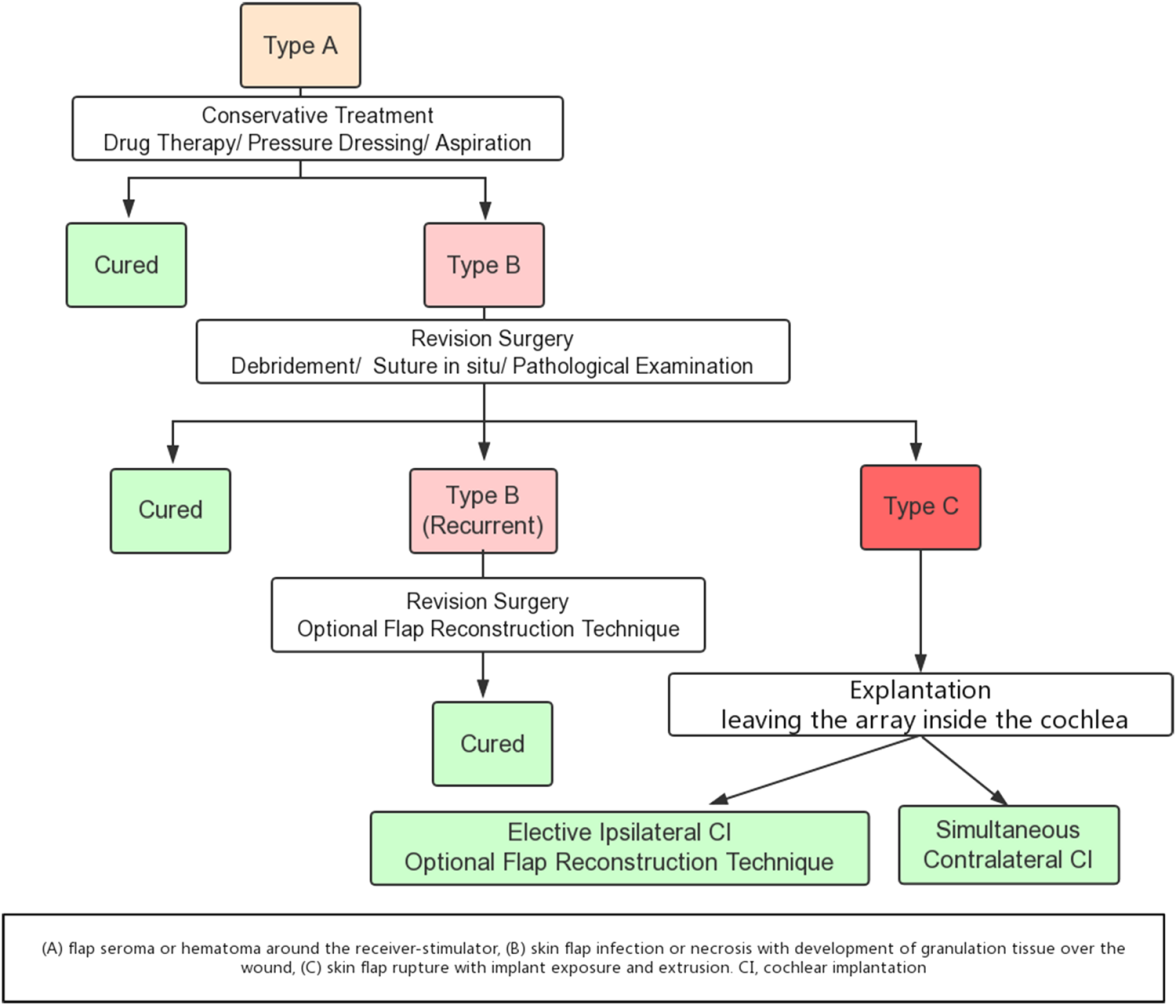
Flow chart of the management of delayed-onset flap complications

All patients should be prescribed prophylactic antibiotics. We adopted treatment strategies appropriate for the various types of flap conditions (see Fig. 2 for a flowchart of patient management).

**Fig. 3:**
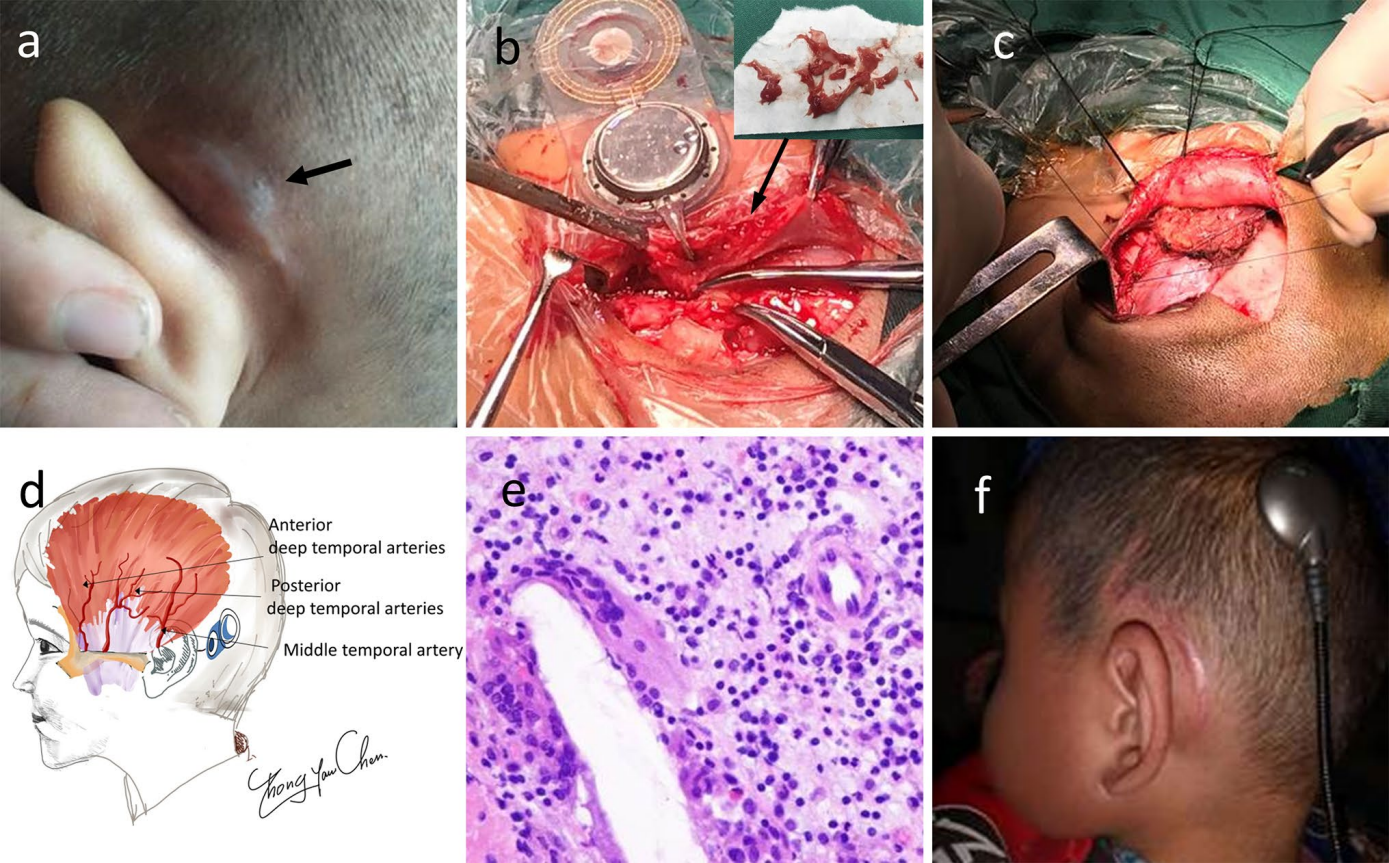
(Patient # 8). **a** The subcutaneous tissue was recurrently swollen with thin and transparent skin. **b** Jelly-like granulation tissue was found encapsulating the device. The implant was repositioned away from the incision. Jelly-like granulation tissue was apparent around the device. Vancomycin powder was distributed around the implantation bed to prevent bacterial biofilm formation. **c** The temporalis myofascial flap technique used to strengthen and repair a subcutaneous defect. Care must be taken not to injure the vessels in the pedicle of the flap. The temporal fascia is freed from the superior temporal line, the temporalis muscle is elevated and partially incised, and the myofascial flap is turned down posteriorly and inferiorly to cover the bony surface and the implant fully. **d** Schematic of the blood supply to the temporalis. **e** Pathological examination revealed nonspecific inflammation, giant phagocytes, and a few eosinophilic and neutrophilic granulocytes. **f** The wound healed well

1. For delayed-onset seroma or hematoma, patients were managed by needle aspiration. A mastoid pressure bandage was applied for about 1 week after the puncture procedure, combined with application of antibiotics and glucocorticoids. Bacterial culture and smear microscopy of contents were conducted. Repeat aspiration was required if swelling did not resolve.
2. In patients who progressed to skin flap defects, timely revision surgery was critical, including thorough debridement of unhealthy inflammatory granulation tissue at the implant site and mastoid, irrigation, and primary suture in situ. Sometimes, the receiver-stimulator needed to be repositioned away from the incision and re-fixed with Prolene sutures. Bacterial and pathological examinations were conducted. The patients were treated with intravenous ceftriaxone at 75 mg/kg for 10 days.
3. For patients in whom revision surgery failed with severe flap rupture and implant device extrusion, explantation ultimately led to wound healing in all cases. Implant removal and contralateral or ipsilateral CI would be considered, leaving the array inside the cochlea, but ipsilateral insertion should be suggested at least 6 months later.
4. Reconstruction of tissue defects using temporalis myofascial flaps. For cases with severe subcutaneous tissue defects, a rotational temporalis myofascial flap may protect the implant. The skin and subcutaneous tissue were elevated in the plane of the temporalis fascia, and an adequate temporalis myofascial flap was harvested whenever possible. To reduce bleeding, we usually injected 100 mL of an adrenaline solution (in saline) during flap preparation, taking care to avoid injuring vessels in the flap pedicle. The temporal fascia was freed from the superior temporal line, and the temporalis muscle elevated and partially incised. The myofascial flap was turned down both posteriorly and inferiorly to ensure full coverage of the bony surface and implant (Fig. 3c, d).

### Statistical analysis

Quantitative variables are presented as means. The Chi squared test was used to compare the effects of incision shape and cochlear implant type and texture on the rate of flap-related complications. A *p* value less than 0.05 was considered statistically significant. All statistical tests were two-sided and were performed using SPSS software (ver. 20.0; SPSS Inc., Chicago, IL, USA).

## Results

In our series, a total of 12 patients (1.48%, 12/811) developed flap complications (Table 2). The delayed-onset flap complication rate after cochlear implantation via a C-shaped incision was 1.57% (12 of 766) and that after implantation through an S-shaped incision 0% (0 of 45) [Chi squared test, *p* = 0.398, difference not significant]. No significant difference in the flap complication rate was observed when the cochlear implants of the different manufacturers and texture were compared (*p* = 0.153, *p* = 0.398, Table 3). The initial implantation procedure was performed in patients ranging in age from 18 to 79 months (median 37.6 months), and the study population consisted of five females and seven males. Flap complications were observed between 2 months and > 10 years after CI. Probable risk factors included acute upper respiratory tract infection (*n *= 2, patients 6 and 8), head trauma (*n * = 2, patients 8 and 11), foreign body reaction (*n * = 3,patients 7, 9, and 12), and strong magnetic coil strength (*n * =  2, patients 3 and 10). There were no obvious predisposing factors in the remaining cases (*n * = 4, patients 1, 2,4, and 5).

**Table 2:** Demographic characteristics of patients with skin flap complications

*n*	Sex	Age at CI (months)	Array/speech processor	Probable cause	Skin flap condition	Delay (months)	Management and outcome			
							Conservative treatment/	Revision surgery	Temporalis myofascial flap technique	Implant removal and re-implantation
1	F	28	CA24RE/Sprint	Unknown	A	122	Cured	–	–	–
2	M	30	CA512/CP910	Unknown	A	13	Cured	–	–	–
3	F	22	CA512/ CP910	Excessive magnetic force	A	26	Cured	–	–	–
4	M	79	Sonata/Opus 2	Unknown	A	49	Cured	–	–	–
5	M	62	Sonata/Opus2	Unknown	A	70	Cured	–	–	–
6	M	41	HR90K 1j /Harmony	URTI	A → B	15	Revision surgery	Cured	–	–
7	F	22	HR90K 1j /Harmony	Foreign body reaction	A → B	1 +	Revision surgery	Cured	–	–
8	M	35	HR90K 1j /Harmony	URTI, trauma	A → B	13	Revision surgery	Cured, twice	**+**	**–**
9	F	36	HR90K 1j /Harmony	Foreign body reaction	A	2	Cured	–	–	–
10	F	42	Concerto/Ronde 2	Excessive magnetic force	B	2 +	Cured	–	–	–
11^a^	M	18	HR90K 1j /Harmony	Trauma	A → B → C	8	Revision surgery	Failed	**+**	Ipsilateral implantation
12	M	36	CA24RE/Freedom	Allergy	C	56	Revision surgery	Failed	–	Contralateral implantation
		Mean: 37.6				Mean: 31.4				

A: flap seroma or hematoma, B: skin flap infection with granulation tissue developed over the wound, C: skin flap rupture with implant exposure and extrusion

*URTI* upper respiratory tract infection

^a^Haemophilus influenzae was found through microbiological culture

**Table 3:** The effects of device type and texture and incision shape on the frequency of delayed-onset flap complications

Delayed-onset flap complications			*p* value
	1 **–**	2 **+**	
Device type			
Cochlear nucleus	449	4	0.153
Advanced bionics	142	5	
Med El	171	3	
Nurotron	37	0	
Incision shape			
“C”	754	12	0.398
“S”	45	0	
Implant texture			
Metal package	754	12	0.398
Non-metal package	45	0	

1 − No delayed-onset flap complications, 2 + delayed-onset flap complications recorded

Most patients first showed delayed scalp seroma or hematoma around the receiver (type A). Flap swelling episodes were encountered in 10 patients at 122, 13, 26, 49, 70, 15, 1, 13, 2, and 8 months postoperatively (patients 1, 2, 3, 4, 5, 6, 7, 8, 9, and 11) (median, 31.9 months). Palpation of the region showed mild pain, soft skin bulges, and difficulty with magnetic adherence of the receiver stimulator. Nine of these ten patients were treated with puncture, pressure mastoid dressing, antibiotics, and antiallergic or glucocorticoid therapy. They were advised not to use the device for a period until the swelling disappeared. The character of puncture fluid content in the swelling was flaxen and transparent serous in two cases, while seven cases had dark red blood. Microscopic analyses of smears from one patient showed full-field erythrocytes and a small number of macrophagocytes (Fig. 1a).

Of the 10 type A patients, 4 (patients 6, 7, 8, and 11) progressed to type B, and patient no. 11 ultimately developed to type C disease with implant extrusion. Patients 10 and 12 were directly diagnosed with lesions of types B and C, respectively. Patient 10 exhibited delayed scalp swelling and ulceration 2 months after surgery due to exposure to a strong magnetic field (Fig. 1b-1). Complete recovery was observed after stopping device use, reducing magnetic strength, and providing early local wound care and medical treatment. Seven of the 12 cases (patients 1, 2, 3, 4, 5, 9, and 10) were healed conservatively with needle aspiration and medical therapy without any surgical procedures.

We performed revision surgery in five cases (patients 6, 7, 8, 11, and 12), wound closure in three (60%; patients 6, 7, and 8) and revision surgery with explantation in two (40%, patients 11 and 12). Explantation led to complete wound healing. Two cases (patients 6 and 7) achieved wound healing and good functional results after a single operation. One case (patient 8, Fig. 3) exhibited repeated flap infections. The wound healed after the first revision surgery. However, the subcutaneous tissue swelled again and the skin became thin and transparent only 1 month after the first revision surgery (Fig. 3a). A second wound exploration was performed. We carefully separated the stimulator from the bone bed, removed jelly-like granulation tissue, and performed mastoidectomy until fresh and healthy soft tissue was exposed (Fig. 3b). Vancomycin powder was applied around the implantation bed to prevent bacterial biofilm formation. The device was repositioned away from the incision using sutures and covered with a local temporalis muscle flap (Fig. 3c, d). Intraoperative nerve remote telemetry (NRT) indicated a good response. Normal healing was observed over 1 year (Fig. 3f).

However, patient 11 developed severe flap necrosis with implant device exposure. Bacterial culture showed the presence of *Haemophilus influenzae* type b (HIB). This patient received explantation and ipsilateral implantation 8 months later. Patient 12 exhibited severe skin decomposition when he visited our clinic 5 years after CI. Despite antibiotic, steroid, and antihistamine administration, wound dehiscence and discharge developed. The first revision surgery failed; flap rupture with device extrusion occurred within 1 month (Fig. 1c-1). Finally, the implant was removed and reimplanted contralaterally. During exploration, hoary soft granulation tissue was found around the device. Histological examination revealed foreign body giant cells and an eosinophilic inflammatory reaction (Fig. 1c-2). The 4-year follow-up showed that the device had been beneficial for the patient.

Rotational temporalis myofascial flaps were adopted in patients 8 and 11, who both recovered well. All cochlear implants were working normally at the final follow-up visits (Table 2).

## Discussion

CI is recognized as a safe and effective therapy for severe to profound hearing loss. Surgical complications are rare in experienced hands but can still occur. In the present study, flap issues occurred in 12 cases (12/811), (1.48%); all compromised the extent of coverage. The type of skin incision showed no relation with the occurrence of flap issues in our study population. There was no obvious difference among the various implant devices in terms of the incidence of flap complications, and neither the incision shape nor cochlear implant texture affected the complication rate (Table 3). Although the incidence is not high, management of skin flap complications remains challenging.

Most flap problems are delayed-onset complications. They may develop within a relatively short period or may be delayed up to several years following surgery. [3] Qin et al. reported 15 seromas around implants, all of which occurred within 1–2 years after CI surgery, while Low reported that hematoma episodes occurred 2–12 years (median, 6 years) after CI. [6, 7] Table 2 shows the onset times for each patient in our study population; the times ranged from 2 months at the earliest to 10 years at the latest with a mean of 31.4 months. Therefore, rigorous follow-up for flap complications should be performed for a long time after surgery.

Flap issues are associated with a range of risk factors, including age at implantation, delayed allergic reactions, poor systemic condition, re-implantation, poor hygiene, acute otitis media, bacterial biofilm formation, upper respiratory infection, head trauma, and strong magnet strength [1, 7–9]. Most of our CI patients were children; flap complications are more common in children [4], who are also susceptible to respiratory tract infections and at higher risk of acute otitis media. Trauma over the receiver is of particular concern in children. Also, the scalps of children are thin and excessive magnetic strength is a serious risk factor for flap necrosis. Poor sanitary conditions can also lead to flap infection [1, 8, 10]. The main issues that we encountered included foreign body reactions (3/12, 25%), strong magnetic attraction (2/12, 16.7%), upper respiratory tract infection (2/12, 16.7%), and a history of head trauma (2/12, 16.7%) prior to swelling. Postoperative flap-related issues caused by foreign body reactions have been reported only rarely [11, 12]. Foreign body reactions are inevitable after implant surgery for various reasons, and the reactions are insidious and unpredictable. Two subjects were allergic to a particular batch of 3–0 silk suture material used for securing the subcutaneous tissue; regional granulomatous inflammation formed 2 months following CI, and the sutures were removed on revision surgery. The patients healed within 2 weeks and have exhibited no further problems since. However, we found no obvious predisposing factors in four patients (33.3%); swelling around the receiver was both sudden and spontaneous. The same situation has also been reported by Gawecki et al. [1] and Catli et al. [8]; some patients lack causative predisposing factors.

Flap-related problems cover a wide range of issues, from flap edema to device extrusion [5]. Most authors have divided skin problems into minor and major flap complications [9, 13, 14]. However, there is no specific classification for flap complications. Based on our experience, according to the various conditions of flap complications, we suggest that flap-related issues can be divided into three types. These classifications embrace flap complications associated with CI surgery and may aid the development of appropriate flap management strategies.

The most common flap complications were delayed seroma or hematoma. If there is no improvement with drug therapy, prompt needle aspiration and a compressive dressing should be considered; needle aspiration can immediately differentiate a seroma or hematoma from pus. Application of a tight dressing for at least 1 week prevents the progression of fluid collection and eliminates the dead space between the receiver body and the flap [7]. In our experience, most type A cases resolved with timely conservative management. However, in patients presenting with progressive swelling or development of further flap-related problems, such as flap necrosis or device extrusion, revision surgery is required in a timely manner to correct the infection. Thorough debridement and CI coverage with healthy, vascularized tissue were the keys to successful operation. In particular, it was necessary to excise jelly-like granulation tissue around the implant and reposition the receiver-stimulator distant from the incision. Exploration of the mastoid cavity and irrigation of the surgical field with antiseptic solution should also be performed. We believe that early detection and treatment improve patient outcomes.

However, the outcome of revision surgery was not guaranteed. After unsuccessful revision surgery, two patients (40%, 2/5) developed skin necrosis with implant exposure, similar to previous reports [1, 6]. Conservative treatment with foreign body retention would have aggravated the patient’s condition. Lack of normal device function is also detrimental to the hearing and speech rehabilitation of children. We advocated explantation of the device, removed the infected flap to allow the infection to resolve and preserved the electrode array inside the cochlea to prevent cochlear obliteration and facilitate future re-implantation. Depending on the hearing state in the contralateral ear and the wishes of the patient, simultaneous explantation and contralateral implantation or selective ipsilateral cochlear re-implantation may be considered at least 6 months later. Two therapeutic options were used in our group; explantation led to complete wound healing and both patients benefited from cochlear re-implantation.

During revision surgery, tissue samples were collected from all patients for pathological examination; this allowed us to perform etiological analysis. It is worth noting that jelly-like granulation tissue was found encapsulating the device in our cases. Histopathological examination of the tissue revealed nonspecific inflammation with foreign-body reactions in granulation tissues. In addition to active phagocytes, scattered eosinophils were also seen, Nadol et al. reported that hypersensitivity or a foreign body giant cell reaction may lead to non-electrical device failures in some patients [15]. Bacterial biofilms that form after infection can lead to failure of revision surgery [16]. Pathogens can colonize the implant surface and induce biofilm formation. Biofilm formation can cause recurrent infections and may even lead to explantation. Although it is difficult to completely eliminate bacterial biofilms, we sprayed vancomycin powder around the implant device after thorough debridement. Higher local drug concentrations inhibit bacterial growth and accelerate wound healing.

Bacteriological examination is essential to ensure appropriate antibiotic treatment. The samples analyzed were derived from puncture fluid, swabs from the infected tissue, and pus. In our study, *Haemophilus influenzae* was found in only one patient through microbiological culture, and no bacteria were found in most cases. As bacterial culture requires time with a low rate of positive results, we thus recommend early, intensive targeted therapy with intravenous antibiotics that cross the blood–brain barrier as the first step in treatment of all patients.

If the implant is exposed, several soft tissue coverage options are available [17]. The temporalis myofascial flap has been introduced in reconstruction of soft tissue defects due to CI extrusion or flap necrosis. The temporalis myofascial flap is an axial flap based on the anterior and posterior deep temporal arteries and the superficial temporal vessels. The temporalis myofascial flap has advantages due to its reliable vascular supply, close anatomical vicinity to the implant insertion site, ease of harvest, adequate bulk, and low morbidity at the donor site. This versatile and reliable myofascial regional flap is very suitable for reconstruction of defects developing after CI [18–20]. In our cases, based on the positions of flap rupture and of the subcutaneous tissue defect in relation to the implant receiver, the opisthotic incision was extended 2 cm to the temporoparietal region. The temporalis myofascial flap reinforces tissues around the implant, thereby reducing tension on sutured skin, enhancing the cure rate, reducing the risk of infection, and promoting wound healing without obvious scarring.

## Conclusions

Delayed onset flap-related complications associated with CI are rare but treatable. Here, we first propose a classification scheme for flap complications, thus a delayed seroma or hematoma, flap infection, necrosis, or rupture, and device extrusion. Early diagnosis is very important and appropriate management should be implemented as soon as possible. An appropriate treatment strategy is required according to the clinical condition of the patient. The temporalis myofascial flap technique is a reliable surgical option that has advantages for CI patients with flap-related problems.
